# Changes in Energy Cost and Total External Work of Muscles in Elite Race Walkers Walking at Different Speeds

**DOI:** 10.2478/hukin-2014-0118

**Published:** 2014-12-30

**Authors:** Wiesław Chwała, Andrzej Klimek, Wacław Mirek

**Affiliations:** 1 Institute of Biomedical Sciences, Department of Biomechanics of University School of Physical Education in Cracow, Poland.; 2 Institute of Biomedical Sciences, Department of Physiology and Biochemistry of University School of Physical Education in Cracow, Poland.; 3 Institute of Sport Sciences, Department of Athletics of University School of Physical Education in Cracow, Poland.

**Keywords:** race walking, energy cost, total energy, maximal oxygen uptake, anaerobic threshold

## Abstract

The aim of the study was to assess energy cost and total external work (total energy) depending on the speed of race walking. Another objective was to determine the contribution of external work to total energy cost of walking at technical, threshold and racing speed in elite competitive race walkers.

The study involved 12 competitive race walkers aged 24.9 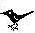 4.10 years with 6 to 20 years of experience, who achieved a national or international sports level. Their aerobic endurance was determined by means of a direct method involving an incremental exercise test on the treadmill.

The participants performed three tests walking each time with one of the three speeds according to the same protocol: an 8-minute walk with at steady speed was followed by a recovery phase until the oxygen debt was repaid. To measure exercise energy cost, an indirect method based on the volume of oxygen uptake was employed. The gait of the participants was recorded using the 3D Vicon opto-electronic motion capture system.

Values of changes in potential energy and total kinetic energy in a gate cycle were determined based on vertical displacements of the centre of mass. Changes in mechanical energy amounted to the value of total external work of muscles needed to accelerate and lift the centre of mass during a normalised gait cycle.

The values of average energy cost and of total external work standardised to body mass and distance covered calculated for technical speed, threshold and racing speeds turned out to be statistically significant (p 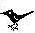
0.001).

The total energy cost ranged from 51.2 kJ.m-1 during walking at technical speed to 78.3 kJ.m-1 during walking at a racing speed. Regardless of the type of speed, the total external work of muscles accounted for around 25% of total energy cost in race walking. Total external work mainly increased because of changes in the resultant kinetic energy of the centre of mass movement.

## Introduction

Substantial aerobic endurance is one of the key factors underlying athletes’ success in endurance sports. In these disciplines, special importance is attributed to maximal oxygen uptake, but particularly to the speed of movement (of running or walking) with exercise intensity at the anaerobic threshold. There are cases, however, that while the variables do not significantly differentiate athletes, their performance is markedly different. This may be due to different energy cost of movement characterising athletes within the same discipline. The value of the variable depends on many factors, mainly on the technique of movement (Klimek and Chwała, 2007).

In the available studies dealing with race walking, energy cost (Klimek and Chwała, 2007) and biomechanical cost (Chwała, 2013) are rarely analysed. Analysis of the literature shows that the problem of estimating the energy cost of athletes was based mostly on the method of heart rate monitoring (Motonaga et al., 2006). Combining the knowledge accumulated by physiology and biomechanics with the available state-of-the art, computerised measurement devices are hard to overestimate. One of them is the possibility of determining the optimal technique of movement that allows energy cost to be kept at a minimum level.

An increase in walking speed sports, on the one hand, results in myocytes increased demand for oxygen to reach the maximum oxygen uptake, on the other hand raises the share of anaerobic processes in the total energy cost.

A reduction in the amount of energy consumed by working muscles directly diminishes their demand for oxygen, thus decreasing the proportion of anaerobic metabolism without affecting the intensity of exercise. This means that the athlete can move faster consuming the same volume of oxygen.

Race walking is an endurance sports event, with a predominance of aerobic metabolism. In the training process of race walkers, different training modes are used for the improvement of technique (technical speed), aerobic capacity (threshold speed), and for competition (racing speed). The assumptions presented above directly apply to the performance of race walkers. The main element of biomechanical cost is energy used to control the displacements of the centre of mass. If its value is low, then the level of total biomechanical cost is also low.

The main component of the biomechanical cost is energy consumed to control the change of the center of mass of the body. Its low value determines the low total biomechanical cost. According to some authors there is a relationship between the physiological and biomechanical cost of the gait (Ortega and Farley, 2005; Umberger and Martin, 2007), therefore Martina et al. (1992) indicate the absence of such a relationship.

Indirect physiological methods mainly calculate the cumulative metabolic cost, whereas the method of calculating the external work performed by the muscles, characterizes the individual technique of control the center of gravity in walking. It is related to the strategy of the movement, aimed at minimizing cost and maximizing energy recovery (Chwała, 2013).

The purpose of the study was to evaluate the energy cost and the total external work (total energy), depending on the speed of race walking. Another objective was to determine the contribution of external work to total energy cost of walking at a technical, threshold and racing speed in elite competitive race walkers.

## Material and Methods

The study was performed on 12 competitive race walkers representing champion and international champion levels. Their age was 24.9 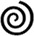 4.10 years, body height 1.80 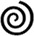 0.68 m, and body mass 69 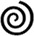 7.06 kg. The mean value of the BMI in the group was 21.26 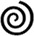 1.81 kg.m-2. The training experience of the participants ranged from 6 to 20 years. Some of them were (1–8) Olympic and World Championships finalists and one was a European Championship medallist. The study protocol was approved by the Local Ethical Committee and was performed in accordance with the Declaration of Helsinki.

The experiment protocol required the athletes to perform typical pre-season training of high volume involving aerobic and mixed exercise (140–180 km of race walking per week). Research was carried out in the morning, in an air-conditioned laboratory at 20–21 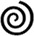
C. The inclusion criteria for athletes, were to be at least of a champion class race walker, provide current medical examination and individual consent to participate in the study.

The aerobic capacity of the participants (maximal oxygen uptake and of the anaerobic threshold) was determined using a direct method involving an incremental exercise test on a treadmill. The first three minutes of the test were a warm up during which the participants walked at 8 km.h-1. Then the walking speed was increased by 1 km.h-1 every three minutes. After consultations with the participants 15 km.h-1 was accepted as the critical speed, after which the treadmill incline was raised 1% to increase exercise intensity. The purpose of this regulation was to prevent participants turning swing phase, that is, from racing to run. The test was continued to volitional exhaustion. The level of the anaerobic threshold was calculated from the dynamics of changes in the respiratory system parameters (the maximum value of FECO2, a significant increase in VE and the minimum value of VE.VCO2-1) (Reinhard et al., 1979).

The purpose of the main component of the experiment was to determine the energy cost of the participants walking with the technical speed vt (when the movement technique can be controlled best), threshold speed – vp (at the anaerobic threshold), and racing speed – vs (used during 20 km race walk events). The threshold and maximum speeds were determined individually for each athlete during the incremental exercise test; the racing speed was established based on the athletes’ performance in 20 km race walk events, depending on their sport level it ranged from 10 to 12 km.h-1. In the exercise test constituting the core of the experiment participants walked with three speeds according to the same protocol: three minutes of rest were followed by 8 minutes of walk with a steady speed (vt, vp, vs,) and by another period of rest until oxygen uptake returned to rest values (the oxygen debt was repaid).

During the exercise test, the respiratory and circulatory systems’ variables (lung minute ventilation - VE, the percentage content of oxygen - FEO2 and carbon dioxide - FECO2 in the exhaled air, minute oxygen uptake – VO2 and the volume of carbon dioxide expelled - VCO2, the breathing quotient - RQ, the breathing equivalent for oxygen - VE.VO2-1 and carbon dioxide - VE.VCO2-1 and heart rate) were recorded for all speeds every 30 seconds.

Exercise energy cost (EE - energy expenditure) was determined by an indirect method based on net oxygen uptake during and immediately after exercise, until all oxygen debt was repaid. The caloric equivalent was used to this end, the value of which was selected in relation to the current ratio between the volume of exhaled carbon dioxide and the volume of oxygen uptake (RER).

The treadmill gait was recorded using a Vicon 250 opto-electronic motion capture system. To enable the acquisition of the measurement data, passive markers were attached to participants’ skin in anatomical points as indicated in the producer’s manual, in conformity with the selected biomechanics model Golem (by Oxford Metrics Ltd). The markers indicated the dimensions and spatial orientation of body segments and the position of joint centres. The measurement data were recorded with a frame rate of 120 fps.

Vertical displacements of the centre of mass (CoM) during a gait cycle were determined using a kinematic method (Eames et al., 1999).

The displacements were used to calculate average values of potential energy changes in relation to the lowest position of the CoM in the gait cycle based on the following formula:

(1) $$\Delta E_{p}=m_{c}g(h_{\text{max}}-h_{\text{min}})\;[\text{J}],$$

where: ΔEp – the average value of changes in potential energy of CoM in the cycle [J]; hmax, hmin – respectively the highest and lowest position of CoM in the cycle [m], mc – body mass [kg].

The resultant CoM velocity was calculated in relation to the axes of the spatial system of coordinates using the relationship:

(2) $$v_{w}=\sqrt{v_{x}^{2}+v_{y}^{2}+v_{z}^{2}}\;[ms-1],$$

where: vx, vy, vz – CoM velocities with respect to particular axes of the spatial system of coordinates.

The resultant CoM velocity average was used to calculate changes in total kinetic energy ΔEk during the gait cycle:

(3) $$\Delta E_{k}=\frac{m\Delta v_{w}{}^{2}}{2}\;\;[\text{J}],$$

where: ΔEk – average value of changes in the resultant kinetic energy of the body during the cycle [J]; mc – body mass of the particular participant [kg]; Δvw – change in CoM velocity.

The value of changes in mechanical energy equivalent to the value of total external energy needed to accelerate and lift the centre of mass in the cycle („total energy”) (Cavagna et al., 2002; Minetti et al., 1994; Schepens et al., 2004) was calculated as a sum of changes in potential energy and resultant kinetic energy of CoM vertical and translational movement according to the following equation:

(4) $$\Delta E_{c}=\Delta E_{p}+\Delta E_{k}\;[\text{J}],$$

where: ΔEc – average value of changes in total energy of CoM.

In the next step, the average changes in potential, kinetic and total energy were standardised by a kilogram of body mass and one meter of distance covered.

To determine the body mass and height of the participants the digital Body Composition Analyzer scale TBF-300 manufactured by the Japanese company Tanita and an anthropometer enabling measurements with accuracy of 0.01 m were respectively used. All exercise test was performed on the Swedish-made treadmill „Cardionics” model 2113. The respiratory system parameters were recorded by a portable ergospirometer Start-2000-M made by MES, which was programmed to take continuous measurements averaged every 30 s. The participants’ heart rate was registered by a recording device connected to the ergospirometer at 15 s intervals.

To capture statistically significant differences between energy cost (EE) and total external work of muscles (ΔEc) for successive walking speeds, the repeated measurement ANOVA and the Tukey’s post-hoc test were applied. The differences between the average values of energy cost (EE) and total external work (ΔEc) were tested for significance by one-way ANOVA, taking the actual walking speed as the dependent variable. To select statistically significantly different pairs of variables in both analyses the Tukey’s post-hoc test was applied.

## Results

In the incremental exercise test, the participants reached maximum oxygen uptake of 67.4 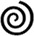 7.25 mL.kg-1.min-1 (4.7 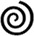 0.81 L.min-1). This result is characteristic of athletes in endurance sports, who can successfully compete in international events (Table 1). The average lung ventilation at which VO2max was reached was 139.7 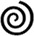 18.8 L.min-1 and the heart rate was 186 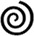 10.7 beats per min-1. Walking speed at the termination of the exercise test (at VO2max) was 15 km.h-1 (4.2 m.s-1, 250 m.min-1), most frequently at a treadmill incline of 1%.

The average energy cost of walking (EE) per 1 minute of exercise for vt was lower by 27.1 kJ.min-1 than in case of vs. Slightly larger differences of EE were established between the technical and threshold speeds (a difference of 35%) than between the threshold and racing speeds (13%). The average values of EE cost calculated for particular walking speeds were significantly different at p 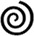
0.001.

An increase was also found in the biomechanical cost of walking calculated as total external work by 0.89 kJ.min-1 for extreme walking speeds. As in case of physiological cost, the cost increased more between the technical speed and threshold speeds (a difference of 16%) than between the threshold and racing speeds (4%). The differences between the average values of total external work at particular walking speeds (kJ.min-1) were statistically significant at p 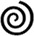
0.001, likewise the differences between the physiological cost of EE and total external work expressed in kJ.m-1 for both the threshold (p 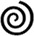 0.05) and racing speed (p 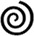 0.005). However, the average values of energy cost obtained with both methods for the technical speed were not significantly different.

The data in Table 2 show that physiological cost standardised by meter of distance covered increased by 59 J.m-1 for extreme walking speeds. This means that the values of variables increased between successive speeds by, respectively, 17% and 4%. EE values calculated for vt and vs, and vt and vp were significantly different (at p 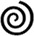 0.005 and p 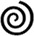 0.01, respectively). There were no significant differences between the threshold speed and the racing speed.

Total external work per one meter of distance covered increased by 20,4 J.m-1, so it increased between successive walking speeds by 18% and 10%, respectively. The average values of total external work per distance covered were significantly different for all walking speeds at p 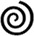
0.001. Similar statistical significance of the differences (p 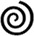 0.001) was obtained when the average values of EE and ΔEc standardized by a meter of distance covered were compared.

The values obtained when energy costs additionally were standardised by one kilogram of body mass were compared and an increase by 0,88 J.m-1.kg-1 was observed. The amounts by which average values increased between successive walking speeds were 17% and 5%, respectively. The increases were significantly different for vt and vp and vt and vs at p 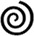 0.005. However, no significant differences between the threshold and racing speeds were noted.

The value of total external work standardised by body mass was higher by 0,3 J.m-1.kg-1 for vs in relation to vt. In relative terms, it increased between the technical speed and the threshold speed by 18%. The difference between the threshold speed and the racing speed was smaller: 10% of the initial value. As in case of total external work standardised by one meter of distance covered, additional standardisation by body mass also produced significant differences for all walking speeds (p 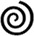
0.001). An analysis of differences between the average values of EE and ΔEc standardised by body mass showed significant differences between all pairs of variables at p 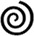
0.001.

## Discussion

Race walking is an endurance sport discipline, so in addition to the perfect walking technique race walkers must show substantial aerobic endurance expressed by maximum oxygen uptake per minute (Schwartz et al., 2006). Compared with results achieved by athletes in other disciplines, including endurance athletes, average VO2max of 67.4 mL.kg-1.min-1 achieved in the experiment does not rank among the highest. In the world-class skiers the variable may even exceed 80 mL.kg-1.min-1 (Bergh, 1982). A similar value has been registered for a four time Olympic gold medallist in race walking during aerobic capacity testing carried out at the Department of Physiology and Biochemistry, of the Academy of Physical Education in Cracow. In this experiment, the participants could effectively compensate for much lower aerobic endurance by being able to walk relatively fast at the anaerobic threshold. An average speed of 3.7 m.s-1 and a comparatively high anaerobic threshold allowed them to perform at a high level during domestic as well as international competitions.

The racing speed of 4.0 m.s-1 the participants achieved is 2.3 times as high as the speed of individually selected fast walk of male non-athletes aged 20–59 years, which is 1.8 m.s-1 on average (Waters et al., 1988). Oxygen uptake in a functional steady state at this walking speed amounted to an average of 18.4 mL.kg-1.min-1 and the heart rate was 124 beats.min-1 (Waters et al., 1983). In participants who walked twice as fast, the minute oxygen uptake was ca. 67.4 ± 7.26 mL.kg-1.min-1 and the pulse rate was 186 ± 18.8 beats per .min-1. Particularly noteworthy is that the participants had lower physiological (aerobic) cost of walking per meter of distance covered (0.17 mL.kg-1.m-1) compared with non-athlete males walking with an almost twice lower speed (0.19 mL.kg-1.m-1).

Total external work of muscles necessary to accelerate and lift the centre of body mass was significantly different from the energy cost EE and its values were distinctly lower. For technical and threshold speeds it accounted for ca 26% and for racing speed for 27% of the energy cost obtained. According to Duff-Raffaele et al. (1996), an analogous proportion of external muscle work in the metabolic cost of a physiological gait at normal speed exceeds 50%. Total external work is exclusive of cost components such as the storage and release of elastic energy (Ishikawa et al., 2005), transfer of energy between the body segments (Bastien et al., 2003), kinetic energy of the rotation of body segments about the biomechanical axis of the joint, or the cost of co-contractions of antagonist muscles and of postural stabilisation. Neptune et al. (2008) state, however, that the total external work of muscles done to lift and accelerate CoM is significantly correlated with metabolic cost.

Total external work of muscles necessary to ensure correct control of CoM displacements shows the nature of the technical gait pattern for particular speeds. Chwała (2013) argues that the amount of energy race walkers can recoup through the inverse pendulum mechanism is small, below 20%. Therefore, minimization of total external work is a proof of an optimised gait technique and indirectly helps reduce energy cost. Significant differences in total external work of muscles in mean values, absolute and standardised by one meter of distance covered and one kilogram of body mass have been found in the experiment (p 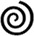 0,001) regardless of walking speed. This reflects that the biomechanical cost increases significantly along with walking speed. Walking speed increasing from technical to racing involved clearly bigger changes in the kinetic energy of the centre of mass related to its acceleration and deceleration. At the same time, changes in potential energy related to the vertical oscillations of the centre of mass remained at a similar level (Table 3).

There have been significant differences between the mean values of physiological cost of walking for all speeds, expressed in kJ.m-1 (p 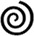
0,001). In addition, we noted significantly different average physiological cost standardized to distance covered (J.m-1) and body mass (J.m-1kg-1), between the technical and threshold speeds as well as the technical and racing speeds (p 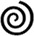 0,005). Significant differences were registered between all considered physiological and biomechanical variables characterizing the energy cost of race walking (p 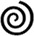
0,001). Mean absolute values of EE and ΔEc (expressed in kJ•min-1) were significantly different for the speed threshold (p 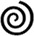 0,05) and home (p 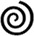 0,005).

Regarding energy cost, the threshold speed and racing speed were not significantly different from each other. On the other hand, a speed increase from threshold to racing had a similar proportion of the technical component in total cost.

Comparing the mean standardized values of the physiological and biomechanical cost we noted significant differences between all the analyzed variables (p 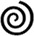
0,001). Mean absolute values of EE and ΔEc (expressed in kJ.min-1) were significantly different for the threshold (p 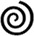
0,05) and racing (p 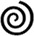 0,005) speed.

A noteworthy finding is that both energy cost and biomechanical cost were much lower in athletes walking at the technical speed, which allows for an optimal gait pattern to be implemented. However, to perform well in race walking events, the speeds must be significantly higher, slightly above the threshold speed, but then the gait becomes more “costly”. Therefore, individual movement technique should be optimised towards minimal vertical oscillations of the centre of mass combined with a smooth passage from the heel-strike to toe-off phase involving only small changes in kinetic energy. The results also clearly indicate that the other components of mechanical energy mentioned above account for a considerable portion of total energy cost. This mean that all factors likely to influence total energy cost must be considered for a movement technique to be correct.

The presented results are important for the practice of sport training, as they enable the optimisation of individual walking technique towards minimum energy cost.

## Conclusions

1. High levels of maximum oxygen uptake and relatively high threshold and racing speeds in the studied athletes showed their high aerobic capacity characterising the ability to perform endurance exercise.
2. The analysis indicated significant differences between the average values of energy costs and total external work calculated for the technical speed, threshold and racing speeds.
3. Total external work of muscles computed in the experiment accounted for around one fourth of total energy cost in race walking regardless of the type of speed.
4. The most important for the total external work increase were changes in the resultant kinetic energy of the centre of mass.
5. In the classification of physical exercise by energy cost race walking with a technical speed is placed in the upper zone of „very heavy” exercise, whereas walking with threshold and racing speeds is recognised as „extremely heavy”.
6. The results of the present study can be used to design training activities aiming at the optimisation of energy cost in race walking.

## Figures and Tables

**Table 1:** Average values of physiological variables during maximal exercise

VO_2_max mL·kg^−1^·min^−1^	VO_2_max L·min^−1^	VEmax L·min^−1^	HRmax sk·min^−1^
67,4 ± 7,25	4,7 ± 0,81	139,7 ± 18,8	186 ± 10,7

VO_2_max – maximum oxygen uptake, VEmax – maximal lung ventilation, HRmax – maxium heart rate

**Table 2:** The average energy cost of walking with technical (v_t_), threshold (v_p_) and racing speeds (v_s_)

V m·s^−1^	Exercise energy cost EE					

	kJ · min^−1^	kcal·min^−1^	J·m^−1^	cal·m^−1^	Jm^−1^kg^−1^	cal·m^−1^kg^−1^
v_t_=3,1±0,19	51,2±10,05	12,3±2,40	267±52,3	63,8±12,52	3,86±0,55	0,92±0,13
v_p_=3,7±0,13	69,2±11,04	16,6±2,64	312±49,7	74,5±11,89	4,51±0,51	1,08±0,12
v_s_=4,0±0,14	78,3±13,01	18,8±3,11	326±54,2	78,1±12,97	4,74±0,62	1,13±0,15

**Table 3:** Average values of ΔE_p_, ΔE_k_ and total external work ΔE_c_ (the biomechanical cost of walking) with technical (v_t_), threshold (v_p_), and racing speed (v_s_)

V m·s^−1^	Biomechanical cost of exercise „total energy”								

	ΔE_p_ kJ·min^−1^	ΔE_k_ kJ·min^−1^	ΔE_c_ kJ·min^−1^	ΔE_p_std_ J·m^−1^	ΔE_k_std_ J·m^−1^	ΔE_c_std_ J·m^−1^	ΔE_p_std_ J·m^−1^·kg^−1^	ΔE_k_std_ J·m^−1^·kg^−1^	ΔE_c_std_ J·m^−1^·kg^−1^
v_t_=3,1±0,19	4,25±0,33	8,91±0,83	13,16±1,25	22,1±2,08	46,4±4,12	68,6±5,13	0,32±0,05	0,67±0,11	0,99±0,14
v_p_=3,7±0,13	4,93±0,42	13,05±1,12	18,00±1,79	22,3±2,06	58,8±4,15	81,1±7,16	0,32±0,06	0,85±0,10	1,17±0,15
v_s_=4,0±0,14	5,14±0,48	16,19±1,37	21,33±1,98	21,4±2,05	67,4±5,20	88,9±8,22	0,31±0,05	0,98±0,13	1,29±0,16
